# Impact of Cadmium Toxicity on Testicular Function: Risk of Male Infertility

**DOI:** 10.3390/life16010181

**Published:** 2026-01-22

**Authors:** Iva Arato, Elena Eugeni, Giuseppe Basta, Tiziano Baroni, Riccardo Calafiore, Francesca Mancuso, Giovanni Luca

**Affiliations:** 1 Department of Medicine and Surgery, University of Perugia, 06132 Perugia, Italy; gius.basta@gmail.com (G.B.); tiziano.baroni@unipg.it (T.B.); riccardo.calafiore@unipg.it (R.C.); francesca.mancuso@unipg.it (F.M.); giovanni.luca@unipg.it (G.L.); 2 International Biotechnological Center for Endocrine, Metabolic and Embryo-Reproductive Translational Research (CIRTEMER), Department of Medicine and Surgery, University of Perugia, 06132 Perugia, Italy; 3 Unit of Internal Medicine, Südtiroler Sanitätsbetrieb, 39012 Merano, Italy; eugeni.elena@gmail.com; 4 Unit of Endocrinology, Andrology and Metabolic Sciences, “Santa Maria” Hospital, 05100 Terni, Italy

**Keywords:** male infertility, cadmium, testicular toxicity

## Abstract

The World Health Organization estimates that about 15% of couples in their adult years in industrialized countries experience infertility, which is described as the inability of a sexually active and non-contraceptive couple to achieve spontaneous pregnancy within a year. Environmental pollution is a significant health concern worldwide and one of the possible risk factors leading to male infertility. Cadmium is a common heavy toxin derived from industrial activities, a ubiquitous environmental pollutant, and can cause severe harm to various organs including the testis. Cadmium toxicity can lead to severe impairment of male germ cells in both rodents and humans, which can result in azoospermia. The negative effects of cadmium on the testicles are caused by its induction of oxidative stress, spermatogenic apoptosis, and testicular inflammation or its detriment to androgenic and sperm cell functions, which damages the vascular endothelium and blood–testis barrier. Overall, this review describes the detrimental impact of cadmium on the testicles and its effect on male infertility. Therefore, by considering recent research findings and identifying future research directions, this review underlines the need to develop new treatments for male infertility related to heavy metal exposure.

## 1. Male Infertility and Environmental Pollutants

Infertility is a rapidly rising issue defined as the inability of a couple of childbearing age to conceive after a year of unprotected intercourse. Infertility affects approximately 15% of couples in industrialized countries [1] and may be caused by factors related to both partners.

**Figure 1:**
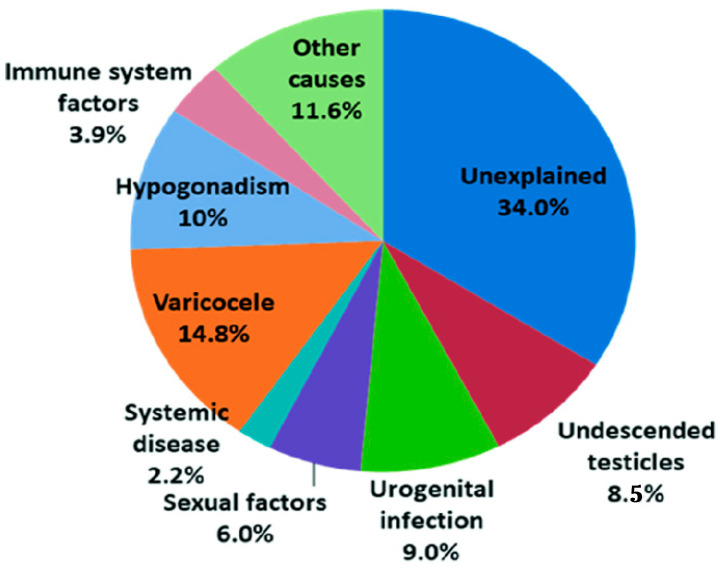
Pie chart showing the etiology of male infertility. More than 30% cases are still unexplained and classified as idiopathic infertility.

Specifically, 30% of cases are indeed driven by a male factor, in addition to a further 20% in which both partners are involved, leading to a value of approximately 50% of cases in which the fertility alteration is clearly linked to the male partner [2]. Unfortunately, despite progress, the etiology of male infertility is still unknown in 30 to 40% of cases, therefore being defined as idiopathic infertility [2,3] (Figure 1).

In these patients, semen analysis often reveals sperm abnormalities (azoospermia, oli-gozoospermia, teratozoospermia, and/or asthenozoospermia) believed to be attributed to multiple factors, such as reactive oxygen species (ROS) production, unknown genetic and epigenetic abnormalities, and endocrine disruption by environmental pollution [4].

The research provides compelling proof that environmental factors significantly impact spermatogenesis and other aspects of male reproductive function.

Studies on both humans and animals have shown that phenols, phthalates, pesticides, air pollution, dioxins, furans, global warming, heavy metals, and tobacco smoke can reduce the fertility of men [5].

An increasing risk to reproductive health is also represented by perfluoroalkyl and polyfluoroalkyl compounds (PFAS); these ubiquitous environmental contaminants have a substantial impact on the phosphorylation of proteins in spermatozoa, in turn affecting critical factors such as sperm motility, viability, and events connected to capacitation [6].

Exposure to environmental pollutants has direct toxic effects on human semen. Furthermore, recent studies suggest that semen can act as an early biomarker for detecting exposure to environmental metal contaminants, providing a valuable tool for assessing the impact of pollution on reproductive health [7].

Studies have demonstrated modifications to sperm nuclear basic proteins (SNBPs), accompanied by a decreased affinity for DNA binding, highlighting the molecular effects of environmental pollutants on sperm integrity [8,9].

The observed deterioration in semen quality reported across multiple studies in recent decades underscores the growing concerns regarding environmental influences, particularly the role of heavy metals as endocrine-disrupting agents.

According to the literature, in terms of the adverse effects of heavy metal exposure on male infertility, cadmium (Cd) exposure is described the most often among the toxic trace elements and thus is the subject of the current review.

Emphasis is placed on its impact on the testes (direct effects on Sertoli cells (SCs), Leydig cells (LCs), spermatozoa, and the blood–testis barrier) and the principal mechanisms involved in testicular damage.

## 2. Sources and Pathways of Cd

Cd is considered a potent mutagen, with high teratogenic and carcinogenic potential [10].

It is released from natural sources, such as volcanic activity, rock erosion, marine water aerosols, and forest fires and is adsorbed in soils and landfills. Cd is a byproduct of the production of other metals such as zinc, lead, or copper; increasing anthropogenic sources are also significant and include batteries, plastic stabilizers, pigments, pesticides, photovoltaic devices, fertilizers, rubber processing, galvanization process, fossil fuel combustion, and waste incineration [11,12] (Table 1).

Due to its non-biodegradable and persistent nature, the amount of Cd in the environment is constantly increasing [12].

The World Health Organization (WHO) has established a tolerable intake level for cadmium at 7 μg/kg of body weight per week. Nonetheless, recent studies indicate that cadmium concentrations capable of inducing biochemical changes and tissue damage may occur at levels currently deemed acceptable by the WHO [13].

The predominant routes through which individuals are exposed to Cd include the consumption of contaminated food or water, inhaling polluted dust, and transdermal absorption. Moreover, a significant source of Cd exposure is cigarette smoking; tobacco has been shown to contain 0.5–5 parts per million of cadmium and one cigarette contains 1–2 μg of Cd (approximately 1.5 μg of Cd), of which almost 10% is inhaled. After consuming just one cigarette, the smoker’s lungs receive about 0.15 μg of Cd [14].

Therefore, the level of Cd in the blood of smokers is 2–3 times higher than in non-smokers [15].

The respiratory system represents the primary route for cadmium absorption, accounting for approximately 13% to 19%, followed by the digestive system, which con-tributes between 10% and 44% [14].

Cd is transported in the blood bound to erythrocytes and proteins (mainly albumin), with a smaller proportion transported by metallothionein (MT) [16].

The liver and kidneys contain roughly 50–75% of the body’s total Cd content [17] (Table 2). The body’s clearance is severely limited, with only approximately 0.007% and 0.009% of the Cd load per day being excreted in the urine and feces, respectively [18]. As a consequence the biological half-life of Cd in humans is approximately 30 years or more [19] (Table 3).

Cd exposure is evaluated via analyzing blood Cd concentrations. The biological limit values (BLVs) for cadmium compounds are set at 1 μg/L in blood and 0.8 μg/L in urine [19].

## 3. Cd’s Impact on Male Infertility

Cd toxicity affects a variety of organs, including the kidney, liver, and lungs [20]. Cd exposure is harmful to both male and female reproductive systems [21,22].

Numerous studies have established that human testes exhibit heightened susceptibility to Cd exposure, leading to toxic effects in male reproductive organs, with pronounced impacts on the testicular tissue and sperm quality. This heightened susceptibility is chiefly attributed to the increased rates of cellular proliferation and metabolic activity of these tissues [23]. Cd is recognized as an endocrine disruptor and reproductive toxicant, adversely affecting the testes even at minimal exposure levels. Prior research has indicated that exposure to Cd can result in testicular tissue damage, decreased testicular mass, compromised testicular function, and reduced androgen synthesis.

Cd penetrates the seminiferous tubules due to damage to endothelial cells, which leads to disruption of the blood–testicular barrier (BTB), subsequently resulting in necrosis and dystrophy of the testicular tissue accompanied by a reduction or complete loss of germ cells [24].

Cd affects two distinct cell populations: LCs, which are responsible for testosterone synthesis, and SCs, which play a crucial role in supporting sperm development [24]. Inflammation and oxidative stress are the principal mechanisms underlying tissue damage caused by cadmium exposure.

Specifically, the production of reactive oxygen species (ROS) is regarded as a principal mechanism driving Cd-induced reproductive toxicity, leading to testicular damage and impairment of spermatogenesis [25]. Furthermore, exposure to Cd has been demonstrated to reduce antioxidant defenses, induce mitochondrial dysfunction, promote lipid peroxidation of cellular membranes, and cause oxidative damage to DNA [26].

Studies have demonstrated that Cd leads to sperm abnormalities in mice and results in the death of male germ cells in both mice and rats [27,28]. Histological analysis on prepubertal rats revealed that Cd exerts detrimental effects on the epithelium of the seminiferous tubules, characterized by cellular desquamation, basal membrane folding, and spermatocyte hypertrophy. Additionally, the histological assessment identified dilated blood vessels and atrophy of LCs within the interstitial tissue [29].

In adult male mice subjected to Cd exposure for seven consecutive days, the secretion levels of sex hormones were markedly affected, specifically luteinizing hormone (LH), follicle-stimulating hormone (FSH), testosterone, and inhibin B [30].

In human studies, occupational exposure to Cd has been associated with apoptosis in fetal germ cells, a decline in semen quality, and oxidative DNA damage in adult human sperm [31,32].

Cd has the capacity to bind to both estrogen and androgen receptors [33].

A meta-analysis performed by Chen et al. synthesized data from 38 studies involving a total of 5070 participants (comprising 3061 males with fertility problems and 2009 healthy controls) and identified a negative correlation between blood Cd levels and total testosterone concentrations [34].

In contrast, results from the 2011–2012 National Health and Nutrition Examination Survey (NHANES) indicated a positive relationship between blood Cd levels and serum testosterone among male subjects [35]. Additionally, Akinloye et al. reported a positive correlation between seminal Cd concentrations and follicle-stimulating hormone (FSH) levels, while no significant association was found between Cd and testosterone levels [36].

These observed variations indicate that Cd negatively influences reproductive hormone regulation; nevertheless, additional investigations are necessary to clarify the specific effects of Cd on the hypothalamic–pituitary–gonadal (HPG) axis.

In summary, Cd detrimentally impacts male fertility through disruption of the hypothalamic–pituitary–testicular axis function [37] and/or by directly inducing gonadotoxic and spermiotoxic damage [38].

## 4. Effects of Cd on Testis

### 4.1. Sertoli Cells

SCs are the only somatic cells within the seminiferous tubules and can be considered the orchestra conductor of spermatogenesis [39]. SCs are responsible for producing multiple factors such as extracellular matrix components, growth factors, and various proteins including transferrin, clusterin, and Stem Cell Factor needed for the successful development of spermatogonia up to the stage of spermatozoa [40,41].

SCs contribute to the formation of the BTB, thereby creating a stable microenvironment essential for the development of male germ cells [42].

During the fetal and neonatal period, SCs are crucial in assembling testis cords.

AMH is an anti-Müllerian hormone also secreted by SCs in the fetus, causing the regression of Müller’s duct [43,44].

The quantity of SCs increases exponentially in both mice and humans throughout fetal development, decelerates following birth, and attains adult levels by the time of puberty [45].

Previous studies mainly conducted in rodents have demonstrated that Cd induces morphological changes in SCs, cell–cell junction alterations, and mitochondrial damage [46,47].

Our group evaluated the effects of Cd toxicity on primary cultures of prepubertal porcine SCs, a bioengineered cell culture system that is a promising experimental model for examining the in vitro effects of toxic substances and heavy metals on male fertility.

The results revealed that even low micromolar concentrations of Cd can affect the functional parameters of superior mammalian SCs, like AMH and inhibin B secretion, which can be deemed sensitive biomarkers of in vitro Cd toxicity in these cells. This data was also supported by the disruption of FSH receptor responsiveness, as measured using Estradiol production, with the induction of SC apoptosis [48].

Zhang et al.’s study demonstrated that Cd has a negative impact on the development of immature SCs in piglet testis, inhibiting their proliferation and leading to apoptosis and DNA damage [49].

Furthermore, Cd has been demonstrated to inhibit the interaction between neonatal SCs and gonocytes in primary murine SC–gonocyte cocultures and to impact the formation of SCs in the fetal and neonatal stages [50].

Li et al. demonstrated that the administration of low doses of Cd results in downregulated gene expression, specifically affecting Desert Hedgehog (Dhh) and the FSH receptor (FSHr) [51].

In an in vitro Sertoli cell–germ cell coculture system, Cd was found to inhibit the p38MAPK signaling pathway, promoting germ cell death and disrupting the interaction between neonatal SCs and germ cells [46].

In pregnant and nursing rats, subcutaneous exposure to Cd (1–2 mg/kg) causes SC vacuolation and deletion of germ cells in the adult seminiferous epithelium.

Additionally, in vitro experiments revealed that Cd affected two actin-regulating proteins, Eps8 and Arp3, thereby damaging the SC actin cytoskeleton; at concentrations ranging from 0.5 to 20 μM, Cd altered the structure of F-actin in human SCs [52].

Rats administered a daily oral dose of 1 mg/kg Cd for a duration of 28 days exhibited significant ultrastructural changes in their adult SCs [53].

**Figure 2:**
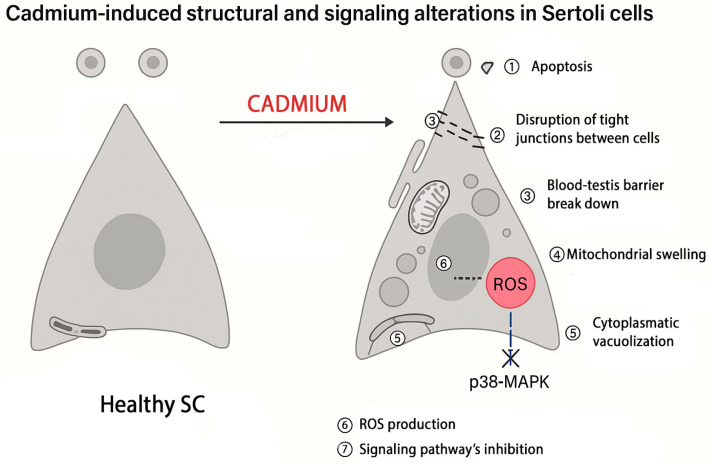
Schematic representation of the harmful effect of cadmium on Sertoli cells.

As a result, studies on animal models concluded that Cd-induced toxicity impacts SCs in a dose-dependent manner by inhibiting p38MAPK signaling, inducing ultrastructural alterations, compromising cell viability, and reducing the levels of hormones such as AMH and INH-B (Figure 2).

### 4.2. Leydig Cells

Within the testes, LCs serve as the primary producers of androgens and contribute significantly to the development of spermatozoa as well as the maintenance of reproductive health. LCs secrete testosterone via the coordinated interaction between the smooth endoplasmic reticulum and mitochondria [54].

Cd exposure causes a reduction in circulating testosterone levels, induces mitochondrial disorganization within LCs, compromises cell viability, elevates lipid peroxidation, causes DNA damage, and impairs the integrity of testicular blood vessels [55].

LCs exhibit a significantly higher sensitivity to Cd compared to SCs for greater expression of cytochrome P450 enzymes and iron cofactors, both of which have a strong affinity for Cd.

This elevated presence of cytochrome P450 enzymes in LCs is crucial because these enzymes are essential for the proper functioning of 17 α-hydroxylase and 17–20 lyase, key enzymes involved in testicular steroidogenesis. Cd exposure disrupts the activity of cytochrome P450 enzymes, potentially impairing steroid hormone production in the testes [56].

LCs are deteriorated by Cd exposure through various mechanisms.

In rat LCs, it has been demonstrated that Cd decreases cell viability and inhibits testosterone synthesis, likely through disruption of mitochondrial function [57].

In a study utilizing the mouse LC line TM3, Cd exposure was observed to significantly disrupt mitochondrial function and elevate both cellular ROS and mitochondrial superoxide levels. The induction of excessive mitochondrial fission is likely a contributing factor to Cd-induced apoptosis in mouse LCs [58].

The expressions of Lhcgr, Scarb1, Star, Cyp11a1, Hsd3b1, Hsd17a1, and Hsd17b3 were markedly downregulated in adult male mice exposed to Cd [59].

In rat testes, Cd treatment caused a decline in the levels of steroidogenic enzymes involved in testosterone synthesis and suppressed the cAMP/protein kinase A (PKA) signaling pathway. This disruption was associated with an increase in premature LCs, which in turn inhibited the proliferation of adult LCs, indicating that Cd exposure negatively impacts testosterone production and LC development [60].

Dihydrolipoamide dehydrogenase (DLD) is a flavin-dependent pyridine nucleotide oxidoreductase localized within the mitochondria of LCs and plays a significant role in LC function. In cultured rat LCs, exposure to Cd resulted in the downregulation of DLD expression alongside a reduction in cAMP levels. These findings indicate that both DLD and cAMP may be critical mediators of the toxic effects induced by Cd in LCs [57].

Collectively, these results indicate that Cd exposure compromises LC function by increasing intracellular ROS, which induce cellular damage through alterations in LC morphology, number, and functional capacity, ultimately leading to cell death.

Additionally, Cd exposure is associated with reduced plasma testosterone levels, disruption of essential signaling pathways, and decreased expression of steroidogenic enzymes.

### 4.3. Spermatozoa

Cd influences the developmental and maturation parameters of spermatozoa, as demonstrated in Table 4.

Spermatozoa contain a cation channel termed CatSper which acts as the principal mediator of intracellular Ca^2+^ influx and promotes various Ca^2+^-dependent physiological processes as sperm motility, viability, and the acrosome reaction. In contrast, the sperm-specific potassium channel (KSper) plays an important role in the hyperpolarization of sperm membrane potential.

It has been shown that the detrimental impact of Cd on the functional activity of these channels contributes to reduced sperm viability and motility, ultimately leading to male infertility [61].

In male C57BL/6 mice, prolonged exposure to Cd—administered both in vitro to sperm samples and orally—adversely affected sperm motility, viability, and the acrosome reaction (AR). Cd-induced impairment of spermatozoa function was attributed to its direct effect on the expression and activity of CatSper channels, leading to disrupted CatSper-mediated calcium permeability and altered CatSper current [62].

Furthermore, a 28-day oral administration of Cd at a dose of 5 mg/kg in rats significantly reduced spermatozoa count, motility, and viability [63].

In vitro exposure of human and mouse spermatozoa to Cd has been shown to significantly impair spermatozoa motility. Zhao et al. demonstrated that prolonged incubation (up to 24 h) of human or mouse sperm with Cd led to a marked concentration- and time-dependent decline in sperm motility. Notably, short-term environmental exposure to Cd (30 min) did not affect sperm motility but significantly decreased the rate of in vitro fertilization [22].

Furthermore, male adolescent Wistar rats exposed to low-to-medium doses of Cd exhibited reductions in sperm motility and epididymal sperm count, with effects dependent on both dose and duration of exposure [64].

In a separate investigation involving Sprague Dawley rats, Yang and colleagues reported that Cd exposure resulted in a decrease in the number of interstitial cells, as well as reductions in round and elongated spermatids (M2). Additionally, Cd induced apoptosis in SCs and undifferentiated spermatids, alongside an increase in inflammatory cell infiltration [55].

Exposure of spermatozoa to Cd was also associated with elevated DNA damage, chromosomal abnormalities, and oxidative stress. Etemadi et al. further observed that Cd treatment of sperm led to chromatin condensation, DNA fragmentation, and alterations in matrix metalloproteinases [64].

**Figure 3:**
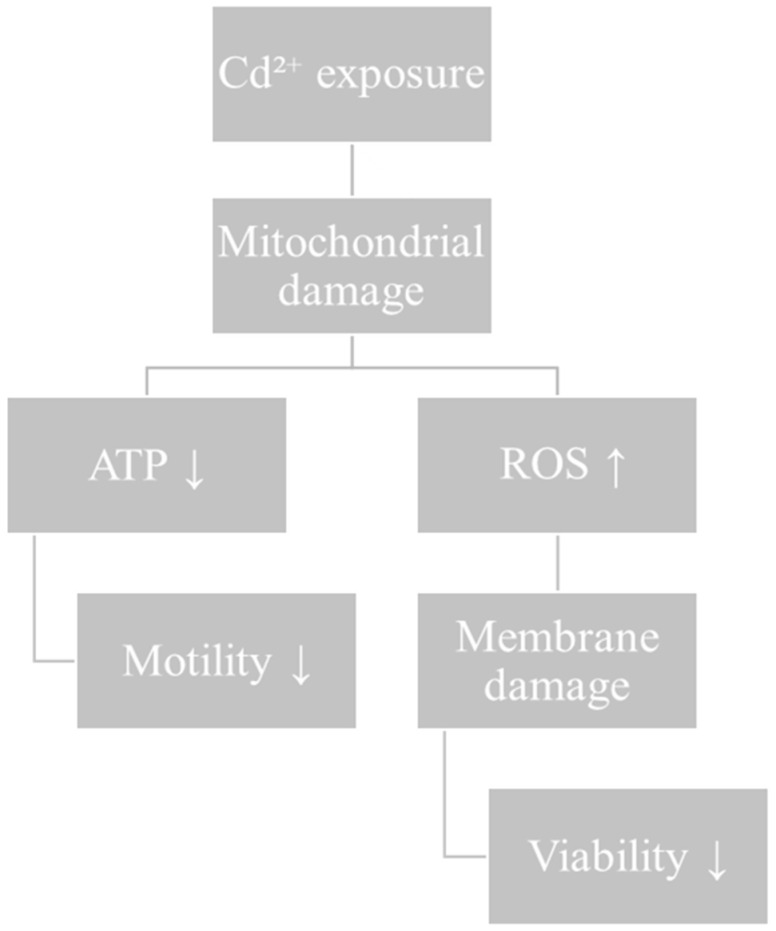
Summary diagram illustrating the direct effects of Cd on sperm functional dynamics. Cd decreases mitochondrial function and ATP production with an increase in ROS. These disruptions lead to functional sperm alterations, including decreased motility and viability.

Cd toxicity in sperm cells is predominantly ascribed to its disruption of the germline differentiation process within the testis. This disruption occurs through the impairment of tight junctions between SCs and the disturbance of cellular redox [65]. Finally, Li et al. showed that Cd exposure promotes the tyrosine phosphorylation of dihydrolipoamide dehydrogenase (DLD), leading to the inhibition of its dehydrogenase activity which subsequently reduces adenosine triphosphate (ATP) synthesis and impairs sperm motility [66]. Overall, it can be stated that Cd exacerbates redox system dysfunction by directly interfering with the mitochondrial respiratory chain or by inducing irreversible oxidative modifications of intracellular antioxidant proteins, as a consequence of excessive ROS generation (Figure 3).

**Table 4:** Cd effects on spermatozoa.

Author (Year)	Model/Species	Main Findings on Sperm/Testis	Proposed Mechanism Pathway
Bashir et al., 2019 [67]; Shi & Fu, 2019 [68]	Mice	Alteration of sperm development and maturation parameters	General Cd-induced toxicity affecting spermatogenesis
Wang et al., 2017 [62]; Zhao et al., 2017 [22]	Human and mouse spermatozoa	↓ Viability, ↓ motility, ↓ acrosome reaction, ↓ fertilizing ability	Interference with Ca^2+^ pathway (CatSper channel)
de Souza Predes et al., 2010 [69]	Rat	Azoospermia induced by low-dose Cd exposure	Testicular toxicity and disruption of spermatogenesis
Sierra-Marquez et al., 2019 [70]	Neotropical fish	Impaired fertilization due to ↓ sperm motility at environmentally accepted Cd levels	Cd-induced motility impairment
Meligy et al., 2019 [71]	Dromedary camels (*Camelus dromedarius*)	Significant differences in pH, concentration, motility, and morphology between fertile and infertile males	Cd-associated seminal alterations
Kollar et al., 2018 [72]; Zhang et al., 2019 [73]	Human and carp	Marked decrease in various sperm parameters	Cd-induced oxidative stress and structural damage
Etemadi et al., 2020 [64]	In vitro (spermatozoa)	Chromatin condensation, DNA fragmentation, apoptotic morphology	Apoptosis induction, chromatin and matrix degradation
Ullah et al., 2023 [74]	Mammal (likely human)	↓ Secretory function of seminal vesicles and prostate → altered semen composition and quality	Endocrine/accessory gland dysfunction

### 4.4. BTB

The BTB in mammalian testes is formed by a specialized junction between adjacent SCs located near the basement membrane within the seminiferous tubules [75].

Damage to the BTB leads to the depletion of germ cells and a reduction in overall sperm count, representing a critical contributor to subfertility and infertility [76].

Cd targets the blood–testis barrier by causing the fragmentation of actin filaments within SCs in both rodent models [77] and human studies [52].

Mechanistic studies have demonstrated that Cd disrupts the BTB in rat testes in vivo by upregulating TGF-β3, which subsequently activates the p38 MAPK signaling pathway [78].

In an in vitro study on rat SCs, Cd influenced the integrity of the BTB in a dose-dependent manner by either preventing the formation of tight junctions or causing their disruption. This effect was mediated through the downregulation of occludin, an integral membrane protein critical to tight junction structure [47].

In rat SCs, an additional mechanism by which Cd disrupts the BTB has been identified. Cd induces the relocalization of occludin and focal adhesion kinase (FAK), a non-receptor protein tyrosine kinase involved in regulation of the BTB.

This study revealed that the administration of Cd caused occludin and FAK to undergo endocytosis; this resulted in their removal from the Sertoli–Sertoli interface and subsequent translocation into the cytoplasm, thereby undermining the structural integrity of the BTB [79].

These data collectively indicate that Cd impairs the BTB in a dose-dependent manner by disrupting the assembly of SCs and downregulating junctional proteins, which leads to reduced cell adhesion and increased vulnerability of the testicular tissue.

## 5. Overview on Mechanisms of Cd Reproductive Toxicity

### 5.1. Oxidative Stress

The generation of ROS at harmful levels transpires exclusively when the antioxidant system fails to maintain homeostatic balance [80].

Cd binding to sulfhydryl groups of ROS scavengers is the primary mechanism that causes an increase in ROS and induction of oxidative stress, which determines an alteration of their regulatory activity, whereas the second mechanism involves interfering with essential ions needed for ROS scavengers’ function, which results in a reduction in glutathione (GSH); both processes converge in the production of ROS, such as superoxide ion, hydrogen peroxide, and hydroxyl radicals [81].

This increase in ROS content induced by Cd leads to an excess of protein oxidation, lipid peroxidation, DNA damage, and ultimately cell death [82].

**Figure 4:**
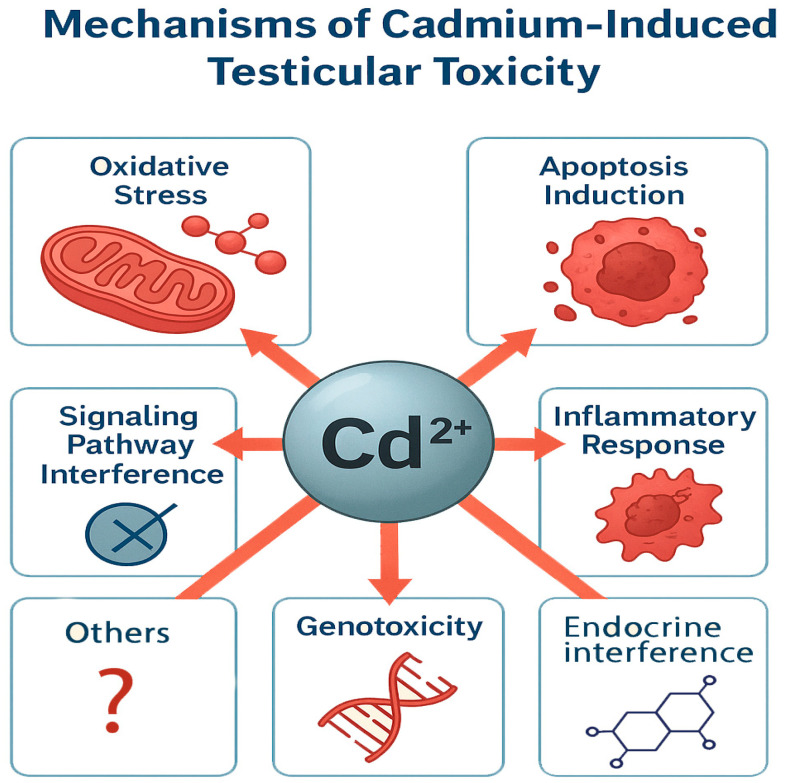
Mechanisms of cadmium toxicity. Some of these have been well established in the literature, while others remain unclear and require further research.

Several studies on different animal species have documented that Cd induces testicular oxidative stress [83]. For instance, the administration of Cd significantly elevated lipid peroxidation activity, the proportion of apoptotic cells, and the activity of antioxidant enzymes, including superoxide dismutase (SOD), glutathione S-transferase (GST), and catalase (CAT). Concurrently, Cd reduces ferric reducing antioxidant power (FRAP) activity in testicular tissue in a concentration- and exposure duration-dependent manner [84]. Wang et al. demonstrated that Cd exposure and the associated oxidative stress impaired the activity of testicular marker enzymes. Additionally, there was a marked reduction in sperm motility and count, attributable to morphological damage within the testicular tissue [85]. In vitro studies involving coculture of germ cells and SCs have shown that Cd increases ROS production and GSH levels, leading to the release of cytochrome C, activation of caspase-3, and subsequent death of SCs [86]. Collectively, these studies indicate that Cd induces oxidative stress by elevating free radical ion concentrations in the testis, which in turn diminishes the levels of antioxidant enzymes such as catalase, superoxide dismutase, glutathione peroxidase, and glutathione reductase (Figure 4).

### 5.2. Induction of Testicular Apoptosis

Apoptosis refers to a form of programmed cell death characterized by a series of distinct morphological and biochemical processes, including cellular shrinkage, fragmentation of DNA, membrane blebbing, chromatin condensation, and the generation of apoptotic bodies [87].

Experimental in vivo studies in animals have shown that Cd disrupts the balance between cell proliferation and apoptosis by downregulating the anti-apoptotic gene B-cell lymphoma 2 (Bcl2) and upregulating the pro-apoptotic genes p53 and Bcl2-Associated X (Bax) [88]. In mouse germ cells, Cd exposure induced apoptosis, decreased B-cell lymphoma extra-large (Bcl-XL), and increased Bax and caspase-3 expression [89].

Further evidence indicated that Cd-induced germ cell apoptosis involves both mitochondrial and endoplasmic reticulum pathways. An essential component of the Fas-mediated caspase-dependent apoptotic pathway, cleaved caspase-8 protein, was found to be produced in increased quantities in the presence of Cd [90].

The Cd-induced cell death process is initiated by mitochondrial membrane depolarization associated with apoptosis and the activation of a DNA damage response.

### 5.3. Induction of Testicular Inflammation

Inflammation is a key mechanism of Cd-induced testicular injury [91]. Studies have reported a significant increase in testicular TNF-α levels following Cd exposure [92]. Elevated expression of *NF-κB*, *COX-2*, and *iNOS* has also been observed, collectively contributing to enhanced oxidative stress [93]. It has been demonstrated that Cd-induced testicular inflammation leads to extensive necrosis and vacuolization of the seminiferous epithelial cells, accompanied by interstitial tissue edema and hemorrhage. These pathological alterations were correlated with a disruption of spermatogenesis [94]. Collectively, these findings suggest that Cd-induced testicular toxicity triggers the production of inflammatory markers which may contribute to the impairment of spermatogenesis and reproductive function.

### 5.4. Genotoxic Effects

Cd is a strong genotoxic agent affecting DNA. The direct damage involves the formation of DNA adducts and inter-strand crosslinks, while oxidative stress indirectly damages DNA. Cd interferes with the activity of key DNA repair enzymes, including DNA polymerases and DNA ligases, leading to the reduced efficiency and fidelity of DNA damage repair [95].

Pant et al. demonstrated that Cd can inhibit DNA ligases, enzymes essential for maintaining the integrity of double-stranded DNA by facilitating the joining of two broken phosphodiester bonds within a DNA strand [96]. Additionally, Cd affects other DNA repair mechanisms, including nucleotide excision repair (NER) and base excision repair (BER), which are responsible for recognizing and rectifying various forms of DNA damage such as those induced by oxidative stress [97].

The disruption of these repair pathways by Cd is closely associated with compromised cellular integrity, leading to cell cycle arrest or apoptosis as a result of accumulated DNA damage.

### 5.5. Interference with Cell Signaling Pathways

Cd has been documented to disrupt calcium homeostasis, calcium signaling, and transcriptional processes [98], as well as modulating the expression of calcium channels [99]. Furthermore, cyclic AMP pathways are associated with calcium metabolism, whereby signaling events in one pathway can induce alterations in the other, with both pathways collectively regulating male reproductive functions [100].

Cd exposure activates several signaling pathways, including the mitogen-activated protein kinase (MAPK), NF-κB, and phosphoinositide 3-kinase (PI3K)/Akt pathways [101]. Cd has also been reported to indirectly affect cAMP metabolism through calcium–calmodulin activation, which stimulates phosphodiesterase activity [102].

In Cd-exposed piglets, increased expression of MAPK pathway components such as p-ERK, p-JNK, and p-p38 mRNA and proteins has been observed in testicular tissue, indicating testicular damage mediated via this pathway [103]. Exposure to Cd induces endoplasmic reticulum (ER) stress through activation of the ATF6 signaling pathway, subsequently leading to apoptosis in vitro. The perturbation of amino acid metabolism, alongside activation of the PERK-p-eIF2α-ATF6 signaling cascade, constitutes a critical mechanism underlying this response [104].

## 6. Potential Protective and Therapeutic Approach

To reduce Cd-induced testicular damage, various compounds with antioxidant properties have been tested—both alone and in combination with another antioxidant. Certain antioxidants including vitamin C, vitamin E, selenium, and glutathione have been shown to confer protective effects on reproductive tissues by mitigating oxidative damage [105].

Resveratrol has also been reported to restore damaged testes after exposure to Cd and to significantly improve the seminal parameter in mice and rats [99].

The consumption of flavonoids, carotenoids, and polyphenol-rich foods counterbalanced the negative impact of Cd. Coenzyme Q10 (CoQ10), recognized for its antioxidant properties, mitigated testicular damage by decreasing the concentrations of titanium (Ti) and Cd within testicular tissue. Additionally, CoQ10 attenuated the expression of TNF-α, which is indicative of its anti-inflammatory effects [106].

Melatonin (MLT) has the ability to protect against Cd toxicity and its effects on testicular function and fertility. However, the cellular and mechanistic aspects of MLT’s effect in testes remain poorly understood.

In one of our works, we have examined the protective effect of MLT on Cd toxicity in porcine prepubertal SCs for the first time. MLT has been demonstrated to counteract the effects of Cd toxicity on antioxidant and detoxification gene expression by regulating redox-sensitive transcription factors and cellular kinases such as NF-κB, c-Jun, MAPK, ERK1/2, and Akt [107]. Notably, these findings are reported for the first time in these cell types and underscore the need for further preclinical and clinical studies to explore the therapeutic potential of MLT in addressing Cd-induced testicular injury and associated fertility impairments.

Chelation therapy and pharmacological agents such as ethylene-diamine-tetra-acetic acid (EDTA), dimercaptosuccinic acid (DMSA), and dimercaptopropane sulfonate (DMPS) improve Cd-induced male infertility. These are absorbed into Cd ions, which are excreted from the body as a result [108].

## 7. Discussion and Conclusions

The high sensitivity of mammalian male gonads to Cd is now well established, and the available data are clear and numerous [26]. Given its widespread presence in the environment, mainly through occupational exposure but also as a result of natural sources, Cd is a global threat to human fertility and the environment [12].

The current review strongly suggests that Cd is a potent testicular toxicant whose harmful effects on the testis result in structural and functional changes that undermine essential spermatogenic processes.

Cd exerts its toxic effects primarily through oxidative stress, disruption of hormonal regulation, and interference with spermatogenesis. The release of ROS produced in the testis under the influence of Cd is identified as a key mechanism leading to sperm damage. Furthermore, Cd disrupts endocrine function by affecting hormone synthesis, secretion, and receptor binding, which negatively impacts spermatogenesis and overall sperm quality [109].

Cd’s toxicity is exacerbated by its ability to mimic zinc ions, disrupting zinc metabolism by substituting zinc in molecular structures such as the zinc-finger domains of DNA-binding proteins. This substitution impairs the function of key enzymes, including superoxide dismutase (SOD), an important antioxidant enzyme, leading to structural changes and reduced enzymatic activity. Since zinc is crucial for testosterone production and spermatogenesis, cadmium’s displacement of zinc significantly contributes to the development of male infertility [23].

Research has elucidated the involvement of the sperm-specific cation channels CatSper and KSper in cadmium-induced male infertility, the role of the Nrf2 signaling pathway in Cd-mediated oxidative stress, and the participation of the MMP/p38 MAPK pathways in cadmium-triggered apoptosis [45].

Nonetheless, its precise mechanism of action is still unclear. More comprehensive research utilizing animal models closely related to humans and representing the actual circumstances of Cd exposure, dosage, and administration routes is needed to completely understand the intricate interplay of various molecular pathways upon Cd exposure associated with male reproductive health and its relationship with epigenetic modifications.

Recent studies highlight the negative correlation between Cd exposure and semen parameters, primarily the count, motility and morphology of spermatozoa [8,9]. Seminal Cd levels may be a useful indicator of male infertility and sperm quality in this context.

Ongoing research highlights the importance of understanding the toxicokinetics of Cd and developing early indicators of exposure, as well as finding approaches to protect against or reduce its harmful effects.

New biomarkers facilitate the development of innovative methods for the early diagnosis and monitoring of Cd exposure, including those that indicate epigenetic changes and oxidative stress.

Antioxidant supplements, chelation therapy, and other compounds have proven effective in protecting against the toxic effects of Cd in various species. However, further investigation is required. Overall, to reduce Cd contamination and develop effective strategies to protect male fertility, it is essential to adopt a multidisciplinary approach that includes rigorous regulatory measures and public health interventions.

However, data in humans has not yet been transferred to clinical application and research into heavy metal damage has not yet become routine, limiting the possibility of collecting data and making progress from a diagnostic and therapeutic point of view. Preclinical research is essential to increase our knowledge in this area for potential future applications.

### Limitations

Research conducted in animal models has substantially contributed to the identification of Cd targets and the definition of possible mechanisms contributing to Cd reprotoxicity, but we must address how humans and animals are more or less susceptible to adverse events and precisely establish the relationship between the experimental doses used in animal studies and realistic human exposure levels.

The analysis of protective strategies is based on emerging evidence, primarily from preclinical studies, and should be interpreted cautiously until validated in large-scale clinical trials. To completely understand the reproductive risks of cadmium exposure, more comprehensive, grounded research is required, as these limitations highlight.

## Figures and Tables

**Table 1:** Major sources of Cd exposure.

Category	Examples	Key Relevance
Natural	Volcanic activity, soil erosion, forest fires	Background exposure
Anthropogenic	Batteries, pigments, fertilizers, smelting	Persistent environmental contamination
Lifestyle	Cigarette smoking	Major contributor to body Cd burden

**Table 2:** Cd distribution in the body.

Step	Description	Reproductive Implication
Blood transport	Bound to erythrocytes, albumin, MT	Controls bioavailability
Major accumulation	Liver and kidney	Long-term internal reservoir
Reproductive tissues	Testis, epididymis, seminal plasma	Direct and indirect sperm toxicity

**Table 3:** Cd toxicokinetics.

Parameter	Description	Fertility Relevance
Absorption	Inhalation > ingestion	Smoking = high risk
Excretion	Extremely low	Progressive accumulation
Biological half-life	20–30+ years	Long-term reproductive risk
Biomonitoring	Blood and urinary Cd	Correlates with sperm endpoints

## Data Availability

No new data were created or analyzed in this study. Data sharing is not applicable to this article.
